# A semi-automated magnetic capture probe based DNA extraction and real-time PCR method applied in the Swedish surveillance of *Echinococcus multilocularis* in red fox (*Vulpes vulpes*) faecal samples

**DOI:** 10.1186/s13071-014-0583-6

**Published:** 2014-12-19

**Authors:** Mats Isaksson, Åsa Hagström, Maria Teresa Armua-Fernandez, Helene Wahlström, Erik Olof Ågren, Andrea Miller, Anders Holmberg, Morten Lukacs, Adriano Casulli, Peter Deplazes, Mikael Juremalm

**Affiliations:** Department of Virology Immunobiology and Parasitology, National Veterinary Institute, Uppsala, Sweden; Institute of Parasitology, Vetsuisse and Medical Faculty, University of Zurich, Zurich, Switzerland; Department of Epidemiology, National Veterinary Institute, Uppsala, Sweden; Department of Pathology and Wildlife Diseases, National Veterinary Institute, Uppsala, Sweden; Department of Biomedical Sciences and Veterinary Public Health, Swedish University of Agricultural Sciences, Uppsala, Sweden; Precision System Science, Stockholm, Sweden; Department of Infectious, Parasitic and Immunomediated Diseases, Istituto Superiore di Sanità, Rome, Italy

**Keywords:** *Echinococcus multilocularis*, Surveillance, Red fox, Diagnostic method, Magnetic capture, Real-time PCR, Faecal samples

## Abstract

**Background:**

Following the first finding of *Echinococcus multilocularis* in Sweden in 2011, 2985 red foxes (*Vulpes vulpes*) were analysed by the segmental sedimentation and counting technique. This is a labour intensive method and requires handling of the whole carcass of the fox, resulting in a costly analysis. In an effort to reduce the cost of labour and sample handling, an alternative method has been developed. The method is sensitive and partially automated for detection of *E. multilocularis* in faecal samples. The method has been used in the Swedish *E. multilocularis* monitoring program for 2012–2013 on more than 2000 faecal samples.

**Methods:**

We describe a new semi-automated magnetic capture probe DNA extraction method and real time hydrolysis probe polymerase chain reaction assay (MC-PCR) for the detection of *E. multilocularis* DNA in faecal samples from red fox. The diagnostic sensitivity was determined by validating the new method against the sedimentation and counting technique in fox samples collected in Switzerland where *E. multilocularis* is highly endemic.

**Results:**

Of 177 foxes analysed by the sedimentation and counting technique, *E. multilocularis* was detected in 93 animals. Eighty-two (88%, 95% C.I 79.8-93.9) of these were positive in the MC-PCR. In foxes with more than 100 worms, the MC-PCR was positive in 44 out of 46 (95.7%) cases. The two MC-PCR negative samples originated from foxes with only immature *E. multilocularis* worms. In foxes with 100 worms or less, (*n* = 47), 38 (80.9%) were positive in the MC-PCR.

The diagnostic specificity of the MC-PCR was evaluated using fox scats collected within the Swedish screening. Of 2158 samples analysed, two were positive. This implies that the specificity is at least 99.9% (C.I. = 99.7 -100).

**Conclusions:**

The MC-PCR proved to have a high sensitivity and a very high specificity. The test is partially automated but also possible to perform manually if desired. The test is well suited for nationwide *E. multilocularis* surveillance programs where sampling of fox scats is done to reduce the costs for sampling and where a test with a high sensitivity and a very high specificity is needed.

## Background

*Echinococcus multilocularis* was detected in Sweden in February 2011 for the first time [1]. Following this finding, extensive monitoring was performed to determine the prevalence and the geographical distribution of the parasite in the country. A total of 2985 hunter shot foxes were analysed for *E. multilocularis*. This first monitoring showed that the parasite was established in three regions of Sweden and that the prevalence was very low, about 0.1% [2]. It was concluded that the parasite is endemic in Sweden and that more information on the geographical distribution of *E. multilocularis* was needed. Based on this, the Swedish Board of Agriculture decided to perform a second large scale monitoring. Although the segmental sedimentation and counting technique (SSCT) [3], a simplification of the sedimentation and counting technique (SCT) was used in the previous monitoring, it was labour intensive as it required handling of the whole carcass of the fox, and the analyses were time consuming. It was concluded that a test more suitable for large scale monitoring was needed. The test should ideally be applied on faecal samples as sampling of fox faeces would simplify collection of samples. The aim of the project would be to develop a sensitive, specific and automatable test suitable for large scale screening in countries where the incidence is very low. Two test strategies have been developed in the past for the diagnosis of *E. multilocularis* in faecal samples: PCR-based detection of *E. multilocularis* eggs or copro DNA in fox faeces [4-8] and coproantigen ELISA (CoA) [9]. The first described method for detection of *E. multilocularis* DNA in fox feaces was published by Bretagne *et al*. [4]. DNA isolation was performed by lysis in potassium hydroxide and subsequent phenol-chloroform extraction and finally a purification step on a matrix followed by conventional PCR. Mathis *et al*. [7] further developed this method by using an alternative method for sample preparation based on the combination of sieving and flotation to concentrate taeniid eggs and thereby removing both inhibitory substances and non-target organisms. Dinkel *et al.* [10], published a real time nested PCR, but the sample size is limited to 0.5 g and the phenol-chloroform extraction method in combination with the extra step of a nested PCR makes the assay less suitable for large scale screening. The CoA has been used for screening purposes in high prevalence areas where foxes with moderate to high worm burdens can be found, but in low prevalence areas the positive predictive value can be low [11,12]. When used in the Swedish surveillance 2000 to 2009, on approximately 300 samples per year, the annual specificity of the CoA varied between 80 to 100% when confirmed with SCT (pers com E Osterman–Lind). As it was foreseen that large scale monitoring in foxes would be repeated it was decided to develop a test suitable for monitoring in low prevalence areas. To achieve an assay suitable for large scale screening, we chose to use fox faeces as the sample type as they are easy to collect, send and store compared to handling a fox carcass. The aim of the present study is to develop a method for the detection of *E. multilocularis* in faecal samples which is sensitive, specific, cost effective, and possible to partially automate for implementation in large scale monitoring.

## Methods

### Faecal samples

A total of 177 foxes shot by hunters during the official hunting season in January/February (*n* = 108) and in October/November 2012 (*n* = 69) in high prevalence areas in Eastern-Switzerland were used in the evaluation. Half of the foxes were analysed fresh or kept at 4°C for no more than three days to collect viable eggs in a part of the foxes for another project, and the other half were frozen at −80°C for at least one week before analysis. The foxes were necropsied at the Institute of Parasitology, University of Zurich and tested with the SCT [13]. Worms were visualized by microscopy and counted. If the sample had more than 100 worms, all the worms were collected and an aliquot was counted to estimate the worm burden of each fox. If samples were autolytic, this was recorded. From each fox a faecal sample was collected from the rectum. After completion of all necropsies the faecal samples (*n* = 177) were weighed and 3 g from each sample were sent to the Department of Virology Immunobiology and Parasitology, National Veterinary Institute, Uppsala, Sweden.

A total of 2158 fox scats from an ongoing national screening for *E. multiocularis* in Sweden (http://www.sva.se) was also used. Fox scats, and in some instances faecal samples from hunter shot foxes were systematically collected from the whole country.

All samples were stored at −80°C for at least 5 days before being analysed with the MC-PCR.

### Magnetic capture probe and PCR (MC-PCR) assay design

The assays were chosen to target the mitochondrial DNA (mtDNA) of *E. multilocularis*. A capture probe, EmFishF, targeting the NADH dehydrogenase 1 (ND1) gene of the *E. multilocularis* mtDNA molecule was designed and ordered (Biomers.net, Ulm, Germany, Table 1). To achieve a melting temperature of more than 80°C, a probe of 120 bases was needed.

**Table 1:** **Primer and probe sequences used in the assay**

EM MGF/fishF	Capture probe target and PCR assay target sequence, cloned into pEX-A vector	AAG AAT TTT TAT TTT CAA AGT CGT AGG TAT ATT GGT TTG TTG GGC GTT TTT TTG TTA ATA ATT TTG GTT ATT ATA TAT TCT TTT ATT TAT GGT AGA TAT TAT AGT GTT AGT TAT AAT AGT GTG CTG CTC ATA AGA GTT TTT GTG TGT TAC ATT GAT AGG AAT ATT GTT GTA ATA TGG TAT TGT TTA GGA CTT AAT AG
Ecanad426bp	12 s segment of *Echinococcus canadensis* (G10 genotype) cloned into pCR2.1 vector	GCT GAT TTG TTG AAG TTA GTA ATT AAG TTT AAG AAT TTT TAT TTC CAG AGC CGT AGG TAT GTT GGT TTA TTT GGT GTT TTG TTG TTG ATA GTT TTG GTT GTG GTG TAT TCG TTT ATT TAT GGT AGA TAT TAT AGA GTT AGT TAT AGT AGG CTT TCT GTG TTA TGA TTT TTA GCT GCT TCT AGA ATT TCT AGG TAT TCT TTG TTG TGT GCT GGT TGG GGT AGC TAT AAT AGT TAT TCT TTT TTA AGG TCG GTT CGA TGT GCT TTT GGA TCT GTT AGG TTT GAA GCT TGT TTT ATG TGT GTG GTT ATT TTT TGC GCT TTA TGT TGT TGT GGG TAT AAT TTA ATT GAT TTT TAT CAT AGT TAT TGG TGA AGT TGA TTA TTA TTC CCA TTA ATT TAT GGG TTA TTC TTG GTG TGT GTG TTG TGT GAG ACT
EmFishF	ND1 capture probe	Biotin TEG-AAGAATTTTTATTTTCAAAGTCGTAGGTATATTGGTTTGTTGGGYGTTTTTTTGTTAATAATTTTGGTTATTATATATTCTTTTATTTATGGTAGATATTATAGTGTTAGTTATAATAGT
EmMGB_F	12 s Forward primer	GTGCTGCTYATAAGAGTTTTTG
EmMGB_R	12 s Reverse primer	CTATTAAGTCCTAAACAATACCATA
EmMGB_P	12 s MGB-probe	VIC-ACAACAATATTCCTATCAATGT-MGB

Primer and probe design of the PCR assay for *E. multilocularis* detection was performed using the oligo design software Allele ID 7 (Premier Biosoft, Palo Alto CA, USA) based on the following sequences available in GenBank [GenBank: JN175268, EU043372, EU043371, AB244598, AB243207, L4955, AB018440, AB031351, AB024424]. The primers target a 77 base pair region of the 12 s gene in the mitochondrial genome of *E. multilocularis*. Primers and probes were checked for specificity in silico using Basic Local Alignment Search Tool, BLAST (National Center for Biotechnology Information, Bethesda MD, USA). The primers were ordered from Eurofins MWG Operon (Ebersberg, Germany) and the MGB probe from Applied Biosystems (Carlsbad, California, USA).

### MtDNA extraction from faecal matter

The following protocol has been modified [14] to suit the specific needs for homogenisation of taeniid eggs and capture of *E. multilocularis* mitochondrial DNA. For each batch of extracted samples, one positive and one negative process control were included to ensure the functionality of the extraction procedure and the real-time PCR assay. The positive control contained 20 000 copies of the synthetic target EM MGB/fishF (Eurofins MWG Operon, Table 1) according to the manufacturer’s estimation, dissolved in 3 ml of TE pH 8.0. The negative control consisted of 3 ml of negative fox faeces.

#### Homogenisation and lysis

For each sample, a 15 ml Sarstedt screw cap tube containing 400 μl or 800 mg of 0.5 mm zirconium oxide beads, 200 μl or 600 mg of 2 mm zirconia beads (BioSpec Products Inc., Bartlesville OK, USA), 9 ml of homogenisation and lysis buffer (100 mM Tris HCl pH 8.0, 5 mM EDTA pH 8.0, 0.2% SDS, 200 mM NaCl) and 3 ml of 5 M NaCl was prepared. Three ml of faecal matter were then added to each tube. Disruption of the taeniid eggs to solubilise the mitochondrial DNA was done using a FastPrep homogenizer (MP Bio, Santa Ana CA, USA) equipped with the TeenPrep 12×15 ml adapter. Each sample was homogenized six times in 60 second runs at maximum speed, 6.5 m/s. The tubes were then centrifuged for 10 min at 3000 g to spin down coarse material. Eight ml of the supernatant was transferred to a new tube.

#### Removal of free biotin

Streptavidine sepharose was used to remove naturally occurring free biotin from the samples. Per sample, 90 μl of high performance streptavidine sepharose (GE Healthcare, Little Chalfont, UK), binding capacity 300 nmol/ml, was washed four times by spinning it down in a VWR Galaxy mini table top quick centrifuge (VWR, Radnor PA, USA) at 6000 rpm/2000 g for a few seconds, taking off the supernatant and washing it with 1 ml of PBS (10 mM Na_2_HPO_4_, 2.7 mM KCl, 1.8 mM KH_2_PO_4_, 137 mM NaCl). The slurry was resuspended in 90 μl of PBS and added to the 8 ml in the tube prepared after homogenisation. The tube was put on a rotator at room temperature at 10 rpm for 45 minutes to let free biotin bind to the streptavidine sepharose beads before centrifugation at 3500 g for 15 minutes to spin down the sepharose. Six ml of the supernatant was thereafter transferred to a new 15 ml tube.

#### Hybridisation of the biotinylated capture probe

In this step, 10 μl of [1 μM] capture probe EmFishF was added to the tube, which was placed in a 98°C water bath to denature the target mtDNA. The tube was transferred to a 55°C shaking water bath at 80 cycles per min for 45 min to allow hybridisation of the capture probe to the target. This was followed by rotation at 10 rpm at room temperature for 15 min.

#### Capture of target DNA

To capture the target DNA, 45 μl of paramagnetic Nordiag Detach streptavidine coated beads (Nordiag, Oslo, Norway), 10 mg/ml, 2.8 μm, binding capacity 650–1350 pmoles/mg beads, were washed 3 times in 1 ml binding and washing buffer (B&W) buffer (5 mM Tris HCl pH 7.5, 0.5 mM EDTA pH 8.0, 1.0 M NaCl) in a 2 ml tube. The beads were pelletized in a DynaMag-2 magnet (Life Technologies, USA) between washes and resuspended in 45 μl of B&W buffer. The washed beads and 1 ml 5 M NaCl solution were added to the tube containing the capture probe/target DNA complex, and put on a rotator at 10 rpm at room temperature for 60 minutes. This step was followed by automated washing of the paramagnetic beads.

#### Automated washing of magnetic beads

To decrease the cost of labour and sample throughput time, the labour intensive step of washing the magnetic beads was automated using the Nordiag Bullet robot (Nordiag ASA, Norway). To be able to handle the large sample volume, two 10 ml 24-well deep well plates were used together with two high power magnet stations. After capture of the target DNA, 48 fifteen ml tubes per run can be placed in the robot. The samples were transferred from the tubes to two 24-well deep well plates where the first two wash steps were performed. The two following washes were performed in a 1.2 ml 96-well deep well plate, and the magnetic beads were finally resuspended in a 96-well PCR-plate in 100 μl of TE-buffer. This plate was then manually placed on a 96-well heating block set to 99°C to melt the captured mtDNA off the capture probe/magnetic bead complex. The plate was then placed in the Bullet standard magnet intended for 96-well plates to pelletize the beads before the TE-buffer containing the template DNA was transferred to a new 96-well plate.

#### Manual washing of magnetic beads

If no robot is available, the washing of beads can also be performed using a manual protocol. The magnetic beads were pelletized using a Dynal MPC-6 magnet (Life Technologies, USA) for 10 minutes before the buffer was removed. The magnetic beads were then resuspended in 1 ml of B&W buffer and transferred to a 2 ml tube before performing two more washes using the Dynamag-2 magnet (Life Technologies, USA) and letting the beads pelletize for 3 minutes in the smaller tubes. After washing the beads, they were resuspended in 100 μl of TE-buffer and placed on a heating block (99°C) for 10 minutes to melt the captured mtDNA off the capture probe/magnetic bead complex. The tubes were then transferred to the small magnet and the bead-free TE-buffer was pipetted to a new 2 ml tube.

#### Minor groove binder (MGB) hydrolysis probe real time PCR and definition of a positive test result

The mastermix consisted of 7.5 μl Ssofast probes supermix (Bio-Rad, USA), 400 nM of each primer EmMGB_F and EmMGB_R, and 133 nM of the hydrolysis probe EmMGB_P. Reactions were run in a total volume of 15 μl of which 2 μl was template DNA retrieved from the DNA extraction. Samples were run in duplicate using an Applied Biosystems 7500 Fast instrument (Life Technologies, USA) in fast mode (ramp rate 5°C per second). The reaction was initiated by 2 minutes at 95°C followed by 48 cycles of 95°C for 5 seconds and 60°C for 30 seconds. Samples were considered positive if at least one of the duplicates displayed a Cq-value equal to or below the selected cut-off point (see below), and the reaction curve exhibited the characteristic exponential curve shape.

#### Evaluation of the analytic sensitivity of the MC-PCR

The analytic sensitivity of the MC-PCR assay was evaluated on a pool of negative fox faeces divided into 35 samples containing three ml of faeces each. These samples were spiked with 1–13 eggs derived from a gravid *E. multilocularis* worm isolated from one of the positive foxes in the Swedish monitoring during 2011. The worm was put in a Petri dish with 0,8% NaCl +0,2% Tween 20 solution and the last proglottid was cut to release eggs and then the eggs were aspirated with the help of a needle. The eggs were counted on a Petri dish before being transferred to tubes for extraction. Due to difficulties in actually transferring the right amount of eggs, the transferred eggs were also counted within the extraction tubes and this was the reason for the variation in numbers of samples spiked with different amount of eggs. The eggs were microscopically examined and counted and no visible parts from the worm attached to the eggs could be detected. Extracted samples were run in duplicate in the PCR according to the protocol described above. The data from this experiment was analysed using the Excel program PODLOD.xls [15] to determine the limit of detection (LOD) at a probability of detection (POD) of 95%.

#### Evaluation of the analytical specificity of the MC-PCR

To test for specificity, one sample each from *Taenia serialis*, *Hydatigera taeniaeformis*, *Taenia polyacantha* and a synthetic plasmid (MWG Eurofins, see Table 1.) containing part of the 12 s sequence from *E. canadensis* (G10, accession number AB745463) were tested using the MGB taqman assay. To check the specificity of the assay, 100 eggs from each sample was used to spike negative fox faeces which was analyzed by the MC-PCR. For the synthetic plasmid containing the *E. canadensis* G10 sequence, about 20 000 copies were added to negative fox faeces. For typing purposes, 100 eggs from each of the samples were homogenized and extracted using the Diasorin Bullet Stool kit (Saluggia, Italy) before performing the conventional multiplex PCR previously described [8]. Thereafter, part of the 267 base pair fragment of the mitochondrial 12 s gene was Sanger sequenced using the primers Cest 3 and Cest 5.

#### Evaluation of the diagnostic sensitivity, specificity, and ROC analysis

The sensitivity of the MC-PCR was calculated using the SCT as the gold standard. Fox samples (*n* = 177) collected in Switzerland were used for this purpose.

The specificity of the MC-PCR was estimated using 2158 samples from Sweden, a country with very low prevalence, approximately 0.1% [2]. Assuming that all MC-PCR positive samples were false positive, a conservative estimate of the specificity was obtained. The 95% confidence intervals of the sensitivity and specificity estimates were calculated. The agreement between the two tests was assessed with the Kappa statistic. Kendall's rank correlation was calculated to measure the strength of dependence between the Cq-values for each duplicate in the MC-PCR and the number of worms detected by the SCT. Only SCT-positive test results where Cq-values were available in both MC-PCR duplicates were included. The mean and range for all Cq-values in both duplicates of the MC-PCR were also calculated for SCT-positive groups with different number of worms. To provide a summary statistic of test accuracy, a receiver-operating characteristic curve (ROC) was plotted. The area under the curve (AUC) was calculated using samples collected in Switzerland that were positive in the SCT (*n* = 93) and samples collected in Sweden considered to be negative (*n* = 2158). Samples considered to be negative in the MC-PCR had no Cq-value but had to be given a numerical value in the analysis. The value of 48 was selected as since this was the total number of cycles run in the PCR. Calculations were done using package epiR, XLConnect and pROC in R v 3.1.1 [16]. A plot of the Cq-values of the SCT positive samples (*n* = 93) and samples collected in Sweden considered to be negative (*n* = 2158) was done using R v 3.1.1 to support the selection of cut-off point. Although two of the 2158 samples most probably were true positives, as both were collected in a known infected area, they were still included since they cannot be proven to be true positives.

## Results

### Analytic sensitivity of the MC-PCR

The analytical sensitivity was determined by spiking negative fox faeces with a known number of *E. multilocularis* eggs (Table 2). All eleven samples spiked with five *E. multilocularis* eggs were found positive and three out of four samples spiked with one *E. multilocularis* eggs were positive. Of the six samples spiked with six eggs, one sample was found negative. The limit of detection (LOD) was estimated to 5.3 eggs with a 95% confidence interval of 2.8-9.8 eggs using the Excel program PODLOD.xls.

**Table 2:** **Results of the analytical sensitivity of the MC-PCR where 35 samples from a pool of negative fox faeces were spiked with 1–13** ***E*** **.** ***multilocularis*** **eggs**

**Number of eggs in the spiked sample**	**Number of samples**	**Number of samples positive in the MC-PCR**
13	1	1
10	8	8
9	5	5
8	3	3
7	1	1
6	6	5
5	8	8
1	3	2

### Analytical specificity of the MC-PCR

The analytical specificity of the MGB taqman assay was tested on fox faeces spiked with eggs from *Taenia serialis* [GenBank:KP127677], *Hydatigera taeniaeformis* [GenBank:KP127678] and *Taenia polyacantha* [GenBank:KP127679] and a synthetic plasmid containing the 12 s sequence from *E.canadensis* (G10), [GenBank:AB745463], which all gave negative results in the MC-PCR. Sequencing of part of the 267 base pair fragment of the mitochondrial 12 s gene confirmed the species of origin for the eggs used in the experiment by using Basic Local Alignment Search Tool, BLAST (National Center for Biotechnology Information, Bethesda MD, USA).

### Evaluation of the diagnostic sensitivity and specificity

Of the 177 faecal samples used to evaluate the MC-PCR, 93 samples originated from SCT-positive foxes and 84 from SCT-negative foxes (Tables 2 and 3). The distribution of worm burden was skewed, ranging from one up to more than 100 000 parasites with a median value of 84 worms. In three highly infected foxes (>10 000 worms), the number of worms was not investigated more precisely. In two of these three foxes only immature worms were detected (Table 3).

**Table 3:** **Validation of the MC-PCR on faecal samples from 177 foxes from Switzerland, using the SCT as gold standard**

**SCT (worm burden)**	**Number of samples**	**MC-PCR positive**	**Cq-values** ^**a**^ **Mean (range)**
0	84	18	33.4 (31.4-36.2)
1 - 10	26	21	33.0 (30.7-35.1)
11 - 100	21	17	31.1 (28.8-33.5)
101 - 1000	25	25	29.5 (27.5-29.4)
1001 - 10 000	15	15	27.9 (25.0-29.4)
10 001–100 000	3	3	26.5 (22.1-29.4)
>100 000^b^	2	0	
>100 000b^c^	1	1	27.9
Total samples	177	100	

^a^Only SCT-positive tests where Cq-values were available were included.

^b^Large amounts of immature worms, impossible to count.

^c^Large amounts of only proglottids, impossible to count.

Using the SCT as gold standard, 82 out of 93 SCT-positive samples were also positive in the MC-PCR, *i.e*. the sensitivity of the MC-PCR was 88.2% (95% CI: 79.8 - 93.9) (Table 3). In foxes with more than 100 worms, the MC-PCR was positive in 44 out of 46 (95.7%) cases. The two MC-PCR negative samples originated from foxes with only immature *E. multilocularis* worms. In foxes with low worm burden, i.e. 100 worms or less, 38 out of 47 (80.9%) faecal samples were positive in the MC-PCR (Table 2). Eighteen of the SCT negative foxes were MC-PCR positive.

Eight foxes were considered to be autolytic. Of these, six foxes had the same result in both tests (three negative and three positive) and two were positive in the MC-PCR and negative in SCT. Immature worms were found in three SCT-positive foxes and two of these were negative and one was positive in the MC-PCR.

The specificity of the MC-PCR was evaluated using fox scats samples from the Swedish screening program. As of March 2014, a total of 2158 samples have been collected and analysed with the MC-PCR and two positive samples have been identified. Both samples were collected in known infected areas, one in the county of Västra Götaland and one in the county of Södermanland. Although both samples are considered to be true positive, we assumed that both samples were false positive in the MC-PCR, in order not to overestimate the specificity. Despite this assumption, the specificity was estimated to be at least 99.9% (CI = 99.7-100).

The agreement between the MC-PCR and the SCT were substantial with a kappa value of 0.670 (p < 0.001). In 29 cases the tests disagreed (Table 2). Eleven samples were positive in the SCT but negative in the MC-PCR. Two of these had only immature worms. The nine remaining samples had an average of 15 worms (range one to 50 worms). Eighteen samples were negative in the SCT but positive in the MC-PCR. Two of these samples were considered to be “autolytic”.

The correlation between the number of worms in the SCT-positive samples and the Cq-values in each of the MC-PCRs run in duplicate was evaluated on 78 of the 93 SCT-positive samples. Fifteen samples where Cq-values were lacking in one or both replicates were excluded. As the number of worms were not normally distributed and as ties existed in the data, the Kendall rank correlation coefficient was used. The correlation coefficients for each of the MC-PCR-duplicates were – 0.37 and – 0.38 respectively with p-values < 0.001. This negative correlation indicates that the Cq-values decrease with an increasing worm burden. The mean and range for all Cq-values of both replicates are detailed in Table 3.

**Figure 1:**
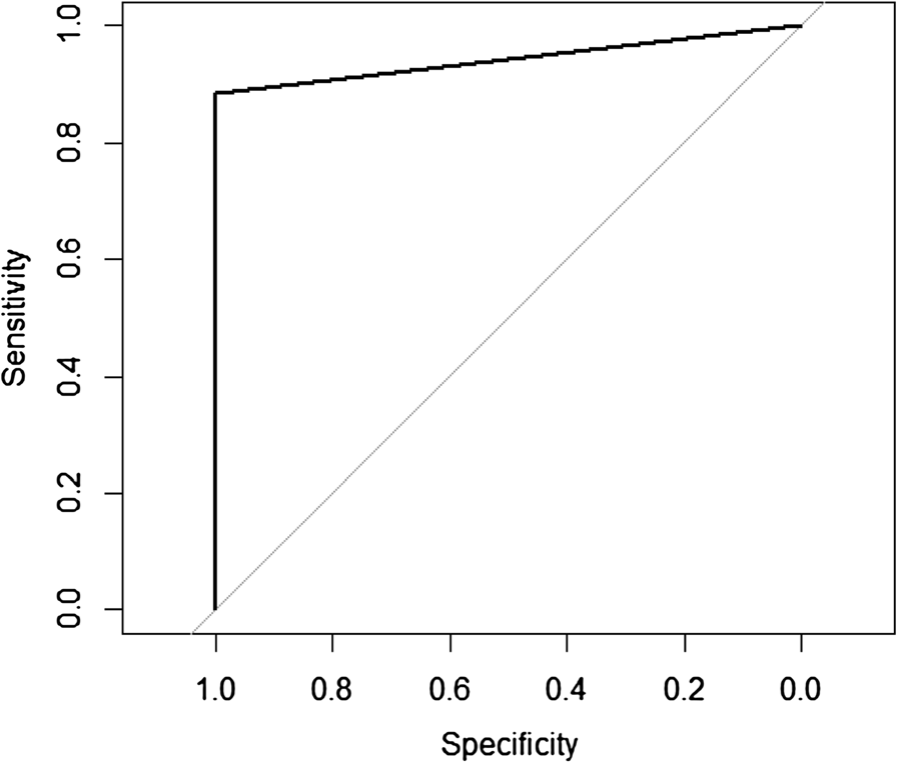
**ROC plot for MC-PCR in 93 SCT-positive fox scats collected in Switzerland and 2158 fox scats collected in Sweden and considered to be negative.**

**Figure 2:**
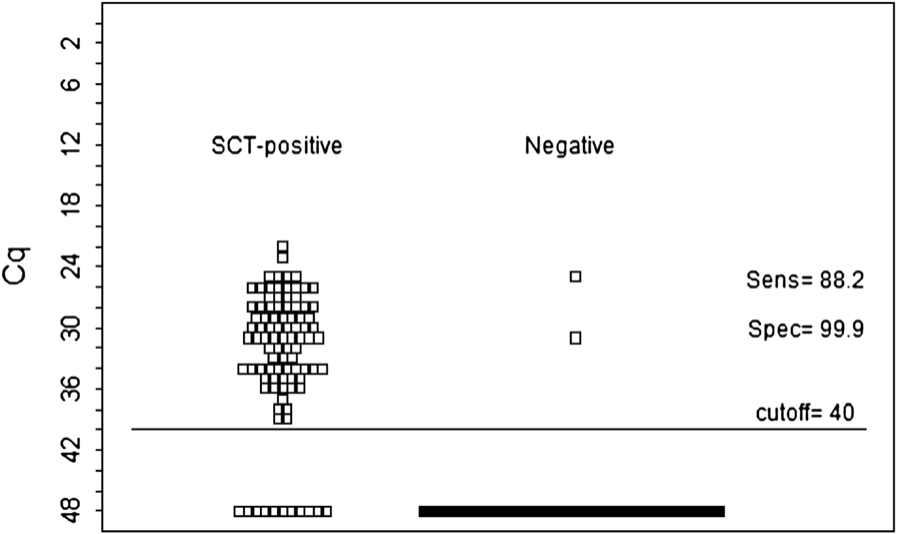
**Plot of Cq-values of negative and posive samples.** Samples without a reaction curve were given the numerical value of 48.

The measurements of test performance expressed as AUC was high, 0.94 (95% C.I. 0.91-0.97). The ROC curve is depicted in Figure 1. The plot of Cq-values of negative and positive samples are detailed in Figure 2. Two of the 2158 samples in the Swedish screening program have Cq-values below 40. Both of these samples are most probably true positives as they both are collected in an infected area.

Eleven of the 93 SCT positive samples from Switzerland had no Cq-value (negative in the MC-PCR). The sensitivity increased up to a cut-off Cq-value of 38.61, the 11 remaining samples had no Cq-value but were given the value of 48 in the analysis. It was therefore concluded that raising the cut-off value above 38.62 does not increase the sensitivity in this dataset, and as the MIQE guidelines [17] advises against using a cut-off value above 40, it was decided to use 40 as cut-off value resulting in a specificity of 0.99 and a sensitivity of 0.88.

## Discussion

The present study demonstrates that the MC-PCR presented in this study can be used as a sensitive diagnostic method for detection of *E. multilocularis* in faecal samples. Previously described PCR methods based on detection of DNA directly in faeces (´copro-DNA PCR´) have until now faced the major obstacle with presence of inhibitory faecal substances [4,7,18]. Even though attempts have been made with commercially available kits tailored for isolation of DNA from faeces, disruption of the taeniid eggs´ keratin layer by homogenisation or alkaline lysis of the sample is essential. Furthermore the capacity to extract DNA directly from faeces from larger samples is limited, usually below 0.5 g which is not sufficient to detect low burden taeniid infections [19]. One approach to overcome the limitation of small sample volume is to perform PCR on isolated taeniids eggs [7]. However, this procedure has its limitations during pre-patent and low patent periods [19], as well as the laborious manual procedure to isolate the eggs. Data on sensitivity compared to previously published protocols is missing since we have not set them all up at our laboratory, and since the data published is not readily comparable between methods. The MC-PCR described in this publication has been tested against a modified version of the egg isolation PCR [21] indicating substantially better sensitivity for the MC-PCR. Since these experiments were performed, the MC-PCR has been improved by changing the reverse primer and automating the wash steps in the extraction procedure.

Regarding the sampling, although not yet described, we do have a red fox specific PCR assay which will be validated for future use to ensure that the faecal samples collected contain DNA from the intended species. Preliminary data indicate that at least 88% of the collected samples contain red fox DNA. A positive result does not necessarily mean that the faeces in fact originates from red fox, merely that the DNA is present in the sample. Also, a negative sample could be red fox faeces where the DNA from the fox was too fragmented to generate a PCR-product.

By applying capture probe based enrichment of *E. multilocularis* DNA after homogenisation of 3 ml faeces, limitations in sensitivity due to inhibitory substances and/or low sample volume can be avoided. The capture probe ensures enrichment of *E. multilocularis* mtDNA onto magnetic beads and allows inhibitory substances and non-target DNA to be washed away. The high analytical sensitivity of the MC-PCR is supported by the results from the spiking experiment (Table 2), indicating that most infected faecal samples containing as few as one *E. multilocularis* egg will in fact be scored as positive, while there is still some risk of missing a positive sample containing as many as six eggs. When spiking buffer with one egg, consistent positive results are achieved (data not published). This indicates that the limiting factor is the homogenization of the eggs in faeces. The heterogeneous nature of fox faecal samples seems to buffer the impact force of the zirconia beads on the eggs during homogenization, preventing disruption. Provided that the mtDNA is in solution, the capture probe extraction method seems to be a very effective way to enrich *E. multilocularis* mtDNA, and the content in one egg is more than enough template for positive results. Since the diagnostic approach is targeting *E. multilocularis* mtDNA within the faeces it should also have the possibility to detect not only eggs but also cells or tissue fragments from disintegrated worm segments. However, the event of shedding cells or tissue fragments from disintegrated worm segments into the faecal matter to be excreted is probably rare.

The mtDNA of *E. multilocularis* is, like in other species, poor in guanine and cytosine (69% AT/31% GC). The low melting temperature of GC poor regions inherently results in long oligonucleotides which in turn are forgiving to mismatches in the sequence of the target DNA. The GC-poor sequence in combination with the fact that *E. granulosus* and other taeniid mtDNA sequences are similar to *E. multilocularis* mtDNA (sequence similarity about 85%) makes species-specific primer and probe design difficult. There are several ways to increase the melting temperature of oligonucleotides apart from increasing their length. One way is to incorporate Locked Nucleic Acids (LNA) [22] in the sequence and another is to synthesise a Minor Groove Binding probe (MGB) [23]. The hydrolysis probe used in the experiments is an unusually long MGB probe containing five mismatches to *E. canadensis* (G10 genotype, the closest taeniid species), ensuring specific detection of *E. multilocularis*. Although the capture probe also captures the *E. granulosus* complex and other *Taenia* spp (data not shown) making it usable for studies focusing on other taeniid species, but the *E. multilocularis* real-time PCR assay ensures the specificity of the assay. This property makes the magnetic capture extraction method also suitable for enrichment and purification of closely related mtDNA involving taeniid species in general.

Using a real time PCR assay is preferable for three reasons. First, there is a high risk of contamination if tubes containing PCR products are opened even if separate laboratory localities are used (conventional PCR, nested conventional PCR and nested real time PCR). Secondly, the step of preparing the second PCR takes extra time (nested conventional PCR, nested real time PCR). Finally, the time and effort required for the detection step is greatly reduced compared to detection by gel electrophoresis (conventional PCR, nested conventional PCR). In some instances a nested PCR will be more sensitive than if a single PCR is used, but considering that inhibition of the PCR reaction usually is the limiting factor when doing extractions directly from faeces, the analytical sensitivity of the amplification step seems to be less important once the problem of inhibition is overcome.

From the evaluation of the diagnostic characteristics of the MC-PCR, we could see that eleven SCT-positive samples were scored as negative in the MC-PCR (Table 4). As the SCT method is based on worm morphology, this test is considered to have approximately 100% specificity (immature *E. granulosus* infections can be excluded in this area for foxes), i.e. no false positive results can occur. Two of the 11 samples only included immature worms and therefore no eggs. Since immature worms do not shed eggs, there will be little or no genetic material for the MC-PCR to detect in these samples. This lack of sensitivity will, of course, apply to all molecular methods using faecal samples. Furthermore, we cannot rule out that the MC-PCR was false negative in the remaining nine samples. The worm burden in these samples was low and the shedding of eggs is maximised around 40 days after infection [24]. Also, a host with gravid *E. multilocularis* parasites does not shed eggs and proglottids continuously.

**Table 4:** **Evaluation of the diagnostic sensitivity of the MC-PCR using the SCT as gold standard in 177 intestinal samples from foxes collected in Switzerland**

		**SCT**		
		+	-	
MC-PCR	+	82	18	100
	-	11	66	77
Total:		93	84	177

Even though SCT is regarded to be the gold standard method for detection of *E. multilocularis* in foxes, its sensitivity has so far not been determined. It was considered unlikely that 18 samples were false positive in the MC-PCR as the PCR method was designed to only detect *E. multilocularis*, and not to cross react with other taeniids. Also, the specificity of the MC-PCR when evaluated on Swedish samples was very high, 99.9% (95% C.I. 99.7-100). In the present study, two of the 18 samples were judged to be autolytic which makes it more difficult to detect worms in the SCT. Furthermore, it cannot be excluded that in high endemic situations harbouring very low worm burden, some positive foxes will be missed in the SCT. Based on this it was concluded that some of the 18 samples were likely false negative in the SCT and true positive in the MC-PCR. In single cases it might be possible that foxes excrete *E. multilocularis* DNA without being infected themselves. For example, in Zurich, only around 10% of rodents infected with *E. multilocularis* have lesions containing protoscoleces [21]. In such cases, the MC-PCR that identifies mitochondrial DNA could be expected to be false positive as the fox is not truly infected. To further support this conclusion, a second real-time PCR using the EVAgreen assay targeting a region in the ND1 gene [20] was used to verify results positive in the MC-PCR but negative in SCT. Fourteen of the 18 SCT negative samples were confirmed by the independent EVAgreen PCR assay, indicating that there in fact was mtDNA originating from *E. multilocularis* present in the sample material. Of the 11 SCT positive/MC-PCR negative PCR, 9 were negative using the EVA green assay.

Samples were regarded positive if at least one of the two replicates gave a Cq-value of 40 or below. One might argue that one out of two should not be enough, and that this kind of result could occur because of contamination. We have investigated the extraction procedure and the automated wash steps by extracting spiked positive and negative samples in a checkerboard pattern and cannot detect any contamination between wells (mean Cq-value 31, standard deviation 0.33 cycles between positive wells). All of the samples in the study, only positive in one of the duplicates, were low positives with high Cq-values (35.4-38.3). Essentially all of them were actually the samples with the highest Cq-values of all of the samples. This is also what we have seen with spiked low positive samples and what we see in other assays where the sample contains very little template. At the limit of detection some replicates will be positive and some will be negative. We do not often see contamination in our molecular diagnostics laboratory and, when we do, we nearly always have an explanation for it (unknown extreme high positive samples, human error or instrument failure such as heated lids failing and allowing PCR product to evaporate out into the lab).

The results in Table 3 show that the mean Cq-value decreases with increased worm burden, although very much less than the “expected” 3.3 cycles per log, as assumed if the shedding of eggs was perfectly proportional to the amount of worms in the intestine. There are several reasons that might explain these results. One reason is related to the fact that the shedding of eggs is not continuous and perfect correlation to worm burden is therefore not to be expected. Eggs are mostly shed during the first period of patency. Around 95% of the total egg production is excreted from foxes during the first 27 days and this period corresponds to only a fraction of the time the mature worm is in the intestine of the host [24]. Also, rare events such as worm fragments being present in the sample will have a large impact on the Cq-value, especially if egg numbers and worm burdens are low. The total spread of Cq-values (min value in the highest mature worm burden class *vs*. max value in the lowest mature worm burden class) is slightly larger than the theoretically expected spread discussed above. Hence, the Cq-values could be considered as an indication but not used as a precise quantification of worm burden. In a recently described qPCR-based method for detection and quantification of *E. multilocularis* in fox faeces, a significant correlation between worm burden and *E. multilocularis* DNA concentration could be seen [6]. However, the results displayed variation between Cq-value and worm burden in a similar way as our results.

In this study we have described a novel approach for enrichment and extraction of *E. multilocularis* DNA from fox faeces. The capture probe based enrichment of *E. multilocularis* DNA minimizes limitations due to sensitivity to inhibitory substances and can be performed on large sample volumes. With automatisation the method is a useful tool in large scale screening and is currently used in a large surveillance program in Sweden.

## Conclusions

As the sensitivity of the gold standard method for the detection of intestinal *E. multilocularis* infections is not known, the true sensitivity of the MC-PCR cannot be calculated. Based on this study it was concluded that the sensitivity of the MC-PCR must be higher than 88%. The specificity was very high, 99.9%. The MC-PCR could therefore be a suitable test for large scale monitoring of fox faecal samples especially in areas where the prevalence of *E. multilocularis* is low. Moreover, the current Regulation (EU 1152/2011) on *E. multilocularis* includes obligations for Member States to implement a pathogen specific surveillance programme aimed at detecting the parasite, thus this method could be used in the future to implement *E. multilocularis* surveillance programmes.

## Abbreviations

MC-PCR: Magnetic capture polymerase chain reaction
SSCT: Segmental sedimentation and counting technique
SCT: Sedimentation and counting technique
CoA: Copro antigen ELISA
mtDNA: Mitochondrial DNA
ND1: NADH dehydrogenase subunit 1 gene
Cq-value: Cycle of quantification
